# Comparing the application of two theoretical frameworks to describe determinants of adverse medical device event reporting: secondary analysis of qualitative interview data

**DOI:** 10.1186/s12913-018-3251-2

**Published:** 2018-06-04

**Authors:** Laura Desveaux, Anna R. Gagliardi

**Affiliations:** 1 Women’s College Hospital, Toronto, Canada; 2 University Health Network, Toronto, Canada

**Keywords:** Equipment and supplies, Physicians’ practice patterns, Determinants, Medical errors, Reporting, Qualitative research

## Abstract

**Background:**

Post-market surveillance of medical devices is reliant on physician reporting of adverse medical device events (AMDEs). Few studies have examined factors that influence whether and how physicians report AMDEs, an essential step in the development of behaviour change interventions. This study was a secondary analysis comparing application of the Theoretical Domains Framework (TDF) and the Tailored Implementation for Chronic Diseases (TICD) framework to identify potential behaviour change interventions that correspond to determinants of AMDE reporting.

**Methods:**

A previous study involving qualitative interviews with Canadian physicians that implant medical devices identified themes reflecting AMDE reporting determinants. In this secondary analysis, themes that emerged from the primary analysis were independently mapped to the TDF and TICD. Determinants and corresponding intervention options arising from both frameworks (and both mappers) were compared.

**Results:**

Both theoretical frameworks were useful for identifying interventions corresponding to behavioural determinants of AMDE reporting. Information or education strategies that provide evidence about AMDEs, and audit and feedback of AMDE data were identified as interventions to target the theme of physician beliefs; improving information systems, and reminder cues, prompts and awards were identified as interventions to address determinants arising from the organization or systems themes; and modifying financial/non-financial incentives and sharing data on outcomes associated with AMDEs were identified as interventions to target device market themes. Numerous operational challenges were encountered in the application of both frameworks including a lack of clarity about how directly relevant to themes the domains/determinants should be, how many domains/determinants to select, if and how to resolve discrepancies across multiple mappers, and how to choose interventions from among the large number associated with selected domains/determinants.

**Conclusions:**

Given discrepancies in mapping themes to determinants/domains and the resulting interventions offered by the two frameworks, uncertainty remains about how to choose interventions that best match behavioural determinants in a given context. Further research is needed to provide more nuanced guidance on the application of TDF and TICD for a broader audience, which is likely to increase the utility and uptake of these frameworks in practice.

**Electronic supplementary material:**

The online version of this article (10.1186/s12913-018-3251-2) contains supplementary material, which is available to authorized users.

## Background

A growing body of research in implementation science has employed classic or implementation science theories or theoretical frameworks to investigate behavioural determinants influencing the use of evidence-based innovations by health care professionals [1]. Given the undesirable prevalence of over-, under- or misuse of innovations and their inconsistent impact on patient outcomes [2], systematic categorization of determinants has been highlighted as a strategy to inform the selection of interventions that best mitigate or address those determinants. The Theoretical Domains Framework (TDF) [3] and the Tailored Implementation for Chronic Diseases (TICD) checklist [4] are two prominent, validated theoretical frameworks that were rigorously developed based on review of the literature followed by international expert consensus. Both facilitate the design of implementation strategies by identifying one or more interventions that may be appropriate for addressing behavioural determinants.

Unfortunately, application of these theoretical frameworks to develop and implement change strategies has proven challenging [5], with an inconsistent impact on health care delivery or patient outcomes [6]. There is a need to improve the selection of behavioural interventions so that they reliably lead to health care improvement. Hence, more insight is needed about the similarities and differences in the content and application of commonly used theoretical frameworks to understand how their use can be optimized when choosing and designing behaviour change strategies.

Previous research has focused on the determinants of implementing practice guidelines, clinical tests or procedures, and quality improvement processes or tools [7, 8]. Despite widespread use of medical devices, little attention has been devoted to understanding determinants of the reporting of adverse events associated with their use. Medical devices include a wide range of health or medical instruments essential for the prevention, diagnosis, cure or management of a disease or abnormal physical condition [9]. Those considered higher risk for adverse medical device events (AMDEs) include orthopedic implants such as hip or knee joints and cardiovascular implants such as pacemakers or implantable cardioverter defibrillators [10, 11]. AMDEs may result from limitations in device design or function, and account for 10% of patient safety incidents in hospitals [12]. Growing concern about AMDEs has led to calls for greater monitoring of outcomes associated with their use [13]. However, registries are not present in every jurisdiction or for every type of medical device. In the absence of systematic data collection, the identification and sharing of information about AMDEs relies on voluntary reporting by physicians.

To learn about AMDE reporting behaviour, we interviewed 22 Canadian physicians who varied by geographical region and career stage; 10 implanted cardiovascular devices and 12 implanted orthopedic devices [14]. When AMDEs arose, they often developed work-around solutions to continue using the same type of device, or they chose to use other comparable devices available on the market. Some participants said they informally shared information about AMDEs with colleagues or industry representatives, however most did not. Determinants of AMDE reporting were identified at the level of the physician (i.e. beliefs about adverse events, device preferences); organization or system (i.e. lack of hospital, national or international reporting policies, systems or incentives); and the device market (i.e. purchasing group contract obligations) [14].

As invasive health care technologies, the characteristics and uses of higher-risk medical devices differ from those of other innovations such as practice guidelines, clinical procedures, or quality improvement processes or tools. Hence, determinants of their use may also differ, providing a unique context within which to study the application of theoretical frameworks for selecting behavioural interventions. The purpose of this study was to (1) categorize determinants of AMDE reporting behaviour that emerged in the primary study using the TDF and TICD; (2) systematically identify interventions that could promote and support AMDE reporting; and (3) compare the determinants and interventions identified by the TDF and TICD as a means of exploring how to optimize the use of those theoretical frameworks in behavioural intervention design. At a practical level, study results will identify interventions that are likely to improve AMDE reporting, thereby optimizing the use and outcomes of higher-risk medical devices. Simultaneously, this work will contribute to the implementation science literature by broadening our understanding of the relevance and application of theoretical frameworks in identifying or describing determinants of innovation use, and selecting corresponding behavioural interventions for change.

## Methods

### Study design

AMDE reporting determinants were mapped to the TDF and TICD to compare determinant domains, determinants and corresponding recommended behavioural interventions. The two authors (LD and ARG) independently mapped the determinants using each framework. LD is an implementation scientist with experience in studying the determinants of physician behavior as it relates to prescribing practices [15], the interdisciplinary management of residents in long-term care [16], and the determinants of patient adherence to recommended treatment following a myocardial infarction [17]. ARG is an implementation scientist with extensive experience in studying determinants of the use of innovations including teamwork in cancer diagnostic assessment programs [18], timely triage and referral of trauma patients [19], the surgical safety checklist [20], guidelines [21] and integrated knowledge translation [22]. ARG has also evaluated the use of theory in assessing barriers of innovation use [23] and in planning behavioural interventions to implement guidelines [24]. ARG had employed the TICD to collect or analyze data in previous studies; she was familiar with the TDF but had not applied it in previous work. LD had not previously applied the TICD but had previous training and experience related to the TDF. This study was based on secondary analysis of qualitative data and did not require ethics approval. However, the University Health Network Research Ethics Board provided ethical approval for the qualitative study that generated data upon which this study is based, and participants of the qualitative study had provided written informed consent prior to being interviewed [14].

### Implementation frameworks

The TDF includes 84 individual determinants across 14 domains (knowledge, skills, social or professional role and identity, beliefs about capabilities, optimism, beliefs about consequences, reinforcement, intentions, goals; memory, attention and decision processes; environment, context and resources, social influences, emotion, behavioural regulation). These domains, and not the individual determinants within them, are linked with 93 behavioural interventions (referred to as behaviour change techniques) across 16 overarching categories [3]. The TICD includes 57 individual determinants grouped in 7 domains (guideline factors, individual health professional factors, patient factors, professional interactions, incentives and resources, capacity for organizational change; social, political, and legal factors), and links individual determinants with one or more of 116 behavioural interventions [4].

### Data collection

AMDE reporting determinants and exemplar quotes that illustrated determinants were acquired from the previously conducted study (Additional file 1) [14]. Methods for the previous study are published elsewhere [14]. In brief, qualitative interviews with physicians that implanted cardiovascular and orthopedic implants were conducted by ARG. Themes reflecting determinants were generated, reviewed and discussed by the entire eight-person research team on four separate occasions to assess thematic saturation, agree upon themes, and interpret data. Themes were organized in the categories of physician beliefs; policies, processes, and systems; and the device market [14].

### Data mapping

Mapping of AMDE reporting determinants to the TDF and TICD was independently performed by LD and ARG. To do this, both used the same version of the TDF [4] and TICD [4] instruments that listed determinant domains, individual determinants (for TICD), and corresponding behavioural interventions. The intent was to undertake naturalistic application of the TDF and TICD that relied solely on the content and guidance provided by the theoretical frameworks themselves. LD and ARG did not review or discuss the content of the TDF or TICD before the independent mapping exercise, nor did they attempt to resolve and reach consensus on discrepancies after mapping. This was an intentional methodological decision to facilitate comparison across mappers using only the frameworks themselves as a guide. AMDE reporting determinants were matched to determinant domains or individual determinants by reading the definitions and examples provided in each framework. LD and ARG each generated a table in which AMDE reporting themes and exemplar quotes were listed along with TDF and TICD domains or determinants thought to be relevant and reflective of the data.

### Data analysis

The two tables reflecting independent mapping were collated to illustrate the TDF and TICD domains or determinants selected by both LD and ARG, and by LD alone and ARG alone. Behavioural interventions corresponding to each domain or individual determinant were extracted from the TDF and TICD and added to the collated table. Domains, determinants and corresponding interventions identified by LD and ARG in the TDF and TICD were enumerated and compared.

## Results

### Mapping of AMDE reporting themes to TDF and TICD

Table 1 summarizes the TDF domains and Table 2 summarizes the TICD determinants selected by one or both mappers.

**Table 1:** Comparison of TDF determinant mapping across mappers

Determinant themes from AMDE study	TDF domains selected by mappers		
	LD	ARG	Domain Match
PHYSICIAN BELIEFS			
AMDEs considered expected or unavoidable and not adverse unless outcomes catastrophic; viewed as more severe in other specialties	Beliefs about consequences	Beliefs about consequences	Yes
	Social-professional role and identity	–	No
AMDEs within 2 years of use were considered unusual	Beliefs about consequences	Beliefs about consequences	Yes
Views about cause of AMDEs confounded by multiple factors	Beliefs about consequences	–	No
	–	Knowledge	No
Incidence of AMDEs has decreased, thus devices were thought to be improved	Beliefs about consequences	–	No
	–	Optimism	No
Sub-total unique or matching domains	2	3	2/7 (28.6%)
POLICIES, PROCESSES or SYSTEMS			
Follow-up of device-related outcomes beyond short-term results done elsewhere	Environmental context and resources	Environmental context and resources	Yes
	Social-professional role and identity	–	No
Devices implanted not recorded in patient records	Environmental context and resources	Environmental context and resources	Yes
No hospital, national or international systems for AMDE reporting	Environmental context and resources	Environmental context and resources	Yes
	–	Reinforcement	No
	Knowledge	–	No
	Behavioural regulation	–	No
Sub-total unique or matching domains	4	2	3/7 (42.9%)
DEVICE MARKET			
Use of specific devices often determined by purchase group contract obligations	Environmental context and resources	Environmental context and resources	Yes
Lack of responsiveness to AMDEs from industry	Reinforcement	Reinforcement	Yes
	Knowledge	–	No
	Optimism	–	No
	Beliefs about consequences	–	No
	–	Environmental context and resources	No
Sub-total unique or matching domains	5	2	2/6 (33.3%)
Total unique or matching domains	6	5	7/20 (35.0%)

**Table 2:** Comparison of TICD determinant mapping across mappers

Determinant themes from AMDE study	TICD domains:determinants selected by mappers		
	LD	ARG	Determinant Match
PHYSICIAN BELIEFS			
AMDEs considered expected or unavoidable and not adverse unless outcomes catastrophic; viewed as more severe in other specialties	Health professional cognitions: expected outcome	Health professional cognitions: expected outcome	Yes
	Health professional cognitions: agreement with the recommendation	–	No
AMDEs within 2 years of use were considered unusual	Health professional cognitions: expected outcome	Health professional cognitions: expected outcome	Yes
	Health professional cognitions: agreement with recommendations	–	No
Views about cause of AMDEs confounded by multiple factors	Health professional cognitions: agreement with the recommendation	–	No
	–	Health professional knowledge and skills: domain knowledge	No
Incidence of AMDEs has decreased, thus devices were thought to be improved	Health professional cognitions: expected outcome	Health professional cognitions: expected outcome	Yes
	Health professional cognitions: agreement with recommendations	–	No
Sub-total unique or matching determinants	3	2	**3/8 (37.5%)**
POLICIES, PROCESSES or SYSTEMS			
Follow-up of device-related outcomes beyond short-term results done elsewhere	Recommended behaviour: observability	Recommended behaviour: observability	Yes
	Health professional cognitions: intention and motivation	–	No
	Health professional behaviour: nature of the behaviour	–	No
	–	Health professional knowledge and skills: knowledge about own practice	No
	–	Health professional behaviour: self-monitoring or feedback	No
	–	Professional interactions: referral processes	No
Devices implanted not recorded in patient records	Incentives and resources: information system	Incentives and resources: information system	Yes
	–	Health professional knowledge and skills: knowledge about own practice	No
	–	Health professional behaviour: capacity to plan change	No
	–	Health professional behaviour: self-monitoring or feedback	No
No hospital, national or international systems for AMDE reporting	Incentives and resources: information system	Incentives and resources: information system	Yes
	Incentives and resources: availability of necessary resources	Incentives and resources: availability of necessary resources	Yes
	Capacity for organizational change: regulations, rules and policies	Capacity for organizational change: regulations, rules and policies	Yes
	Health professional knowledge and skills: domain knowledge	–	No
	–	Health professional cognitions: intention and motivation	No
	–	Health professional behaviour: self-monitoring or feedback	No
	–	Incentives and resources: non-financial incentives and disincentives	No
	–	Incentives and resources: quality assurance and patient safety systems	No
	–	Capacity for organization change: monitoring and feedback	No
Sub-total unique or matching determinants	7	11	5/19 (26.3%)
DEVICE MARKET			
Use of specific devices often determined by purchase group contract obligations	Health professional behaviour: capacity to plan change	Health professional behaviour: capacity to plan change	Yes
	Capacity for organizational change: regulations, rules and policies	–	No
	–	Incentives and resources: financial incentives and disincentives	No
	–	Capacity for organizational change: mandate, authority and accountability	No
	–	Social, political and legal factors: economic constraints on the health care budget	No
	–	Social, political and legal factors: contracts	No
Lack of responsiveness to AMDEs from representatives or manufacturers	Health professional cognitions: expected outcome	–	No
	–	Health professional cognitions: intention and motivation	No
	–	Health professional behaviour: self-monitoring or feedback	No
	–	Social, political and legal factors: influential people	No
Sub-total unique or matching determinants	3	8	1/10 (10.0%)
Total unique or matching determinants	10	19	9/37 (24.3%)

#### All themes were successfully mapped to both frameworks

All AMDE reporting themes (noted in *italics* throughout the manuscript) were directly and clearly addressed by both frameworks, and therefore mapped to one or more TDF domain and TICD determinant. For example, the theme *‘AMDEs were considered unexpected or unavoidable’* aligned with the TDF domain of ‘Beliefs about consequences’ and the theme *‘Lack of responsiveness to AMDEs from industry’* was readily mapped to the TDF domain of ‘Reinforcement’. Similarly, the theme *‘No hospital, national or international systems for AMDE reporting’* was readily mapped to the TICD determinant ‘Incentives and resources: information system’ and *‘Use of specific devices often determined by purchasing group contracts obligations’* was mapped to the TICD determinant ‘Health professional behaviour: capacity to plan change’.

#### A range of domains and determinants were identified

AMDE reporting determinants were mapped to multiple domains and determinants, revealing the interplay of multi-level determinants that influence AMDE reporting, in addition to the complexity of applying the TDF and TICD. In part this was because the previous study [14] identified that physician, organizational, system, and market level factors influenced whether and how physicians reported AMDEs. This was compounded by the reality that AMDE reporting themes often mapped to more than one domain or determinant. For example, the theme *‘No hospital, national or international systems for AMDE reporting’* mapped to 4 different TDF domains (Environmental context and resources, Reinforcement, Knowledge, and Behavioural regulation). The same theme mapped to 5 different TICD domains, representing 9 unique determinants [Incentives and resources (4 determinants): information system, availability of necessary resources, non-financial incentives and disincentives, and quality assurance and patient safety systems; Capacity for organizational change (2 determinants): regulations, rules, and policies, and monitoring and feedback; Health professional knowledge and skills (1 determinant): domain knowledge; Health professional cognitions (1 determinant): intention and motivation; Health professional behaviour (1 determinant): self-monitoring or feedback].

Across both mappers, themes relating to physician beliefs were mapped to 4 unique TDF domains, while organizations or systems and device market were each mapped to 5 unique domains. Overall, the TDF identified 7 unique domains across all AMDE reporting themes. Using the TICD, physician beliefs themes were mapped to 3 unique determinants; policies, processes or systems themes were mapped to 14 unique determinants; and device market themes were mapped to 10 unique determinants. Overall, the TICD identified 21 unique determinants across all AMDE reporting themes.

#### Domains and determinants were convergent across themes

Although AMDE reporting themes were identified at the physician, organization or system, and device market levels, selected domains or determinants were often mapped to multiple themes. For example, the TDF domain ‘Beliefs about consequences’ was applied across multiple themes pertinent to physician beliefs and device market (Table 1). Similarly, the TICD determinant ‘Health professional cognitions: expected outcome’ was applied across multiple themes pertinent to physician beliefs and device market (Table 2).

#### Comparison across mappers

The two mappers differed in the number and domains or determinants matched to AMDE reporting themes, revealing the subjectivity inherent in the mapping process (Tables 1 and 2). For example, both applied the TDF domain ‘Environmental context and resources’ to the theme *‘No hospital, national or international systems for AMDE reporting’*. For the same theme ARG also chose the TDF domain ‘Reinforcement’ and LD also chose the TDF domains ‘Knowledge’ and ‘Behavioural regulation’. For the same theme, both mappers applied the TICD determinants ‘Incentives and resources: information system’, ‘Incentives and resources: availability of necessary resources’ and ‘Capacity for organizational change: regulations, rules and policies’. LD also chose the TICD determinant “Health professional knowledge and skills: domain knowledge” and ARG also chose the TICD determinants ‘Capacity for organizational change: monitoring and feedback’, ‘Health professional cognitions: intention and motivation’, ‘Health professional behaviour: self-monitoring or feedback’, ‘Incentives and resources: non-financial incentives and disincentives’ and ‘Incentives and resources: quality assurance and patient safety systems’. Overall LD applied more TDF domains and fewer TICD determinants compared with ARG, potentially reflecting their individual familiarity with the respective frameworks. For all 20 TDF domains selected across both mappers for all themes, there were 7 (35.0%) matches across both mappers. For all 37 TICD determinants selected across both mappers for all themes, there were 9 (24.3%) matches across both mappers. Thus the proportion of discrepancies across mappers was relatively consistent across the application of both frameworks.

#### Comparison across theoretical frameworks

Table 3 summarizes the TDF domains and TICD determinants chosen by one or both mappers for each AMDE reporting theme. A greater number of TICD determinants were applied overall across themes and mappers compared with TDF domains. This could be attributed to the level of the specificity corresponding to intervention identification (domains for the TDF and determinants for the TICD) or the focus of the frameworks themselves. The TDF largely focuses on determinants of individual behaviour while the TICD offers determinants at the individual, organization or system, and market levels, thus better aligning with the multi-level nature of determinants contributing to AMDE reporting. However, several TDF domains were similar in meaning to TICD determinants, albeit identified by different labels. For example, themes relating to physician beliefs were mapped to the TDF domain ‘Beliefs about consequences’ and the TICD determinant ‘Health professional cognitions: expected outcome’ and policies, processes or systems themes were mapped to the TDF domain ‘Environmental context and resources’ and the TICD determinant ‘Incentives and resources: information system’. Matching of TDF domains and TICD determinants was apparent across all themes and levels.

**Table 3:** Comparison of determinant mapping across theoretical frameworks

Determinant themes from AMDE study	TDF domains selected		TICD domains:determinants selected		Apparent match in underlying meaning
	Both mappers	One mapper	Both mappers	One mapper	
PHYSICIAN BELIEFS					
AMDEs considered expected or unavoidable and not adverse unless outcomes catastrophic; viewed as more severe in other specialties	Beliefs about consequences	Social-professional role and identity	Health professional cognitions: expected outcome	Health professional cognitions: agreement with the recommendation	Yes (expected outcome)
AMDEs within 2 years of use were considered unusual	Beliefs about consequences	–	Health professional cognitions: expected outcome	Health professional cognitions: agreement with the recommendation	Yes (expected outcome)
Views about cause of AMDEs confounded by multiple factors	–	Beliefs about consequences, Knowledge	–	Health professional cognitions: agreement with the recommendation, Health professional knowledge and skills: domain knowledge	Yes (knowledge)
Incidence of AMDEs has decreased, thus devices were thought to be improved	–	Beliefs about consequences, optimism	Health professional cognitions: expected outcome	Health professional cognitions: agreement with the recommendation	Yes (expected outcome)
POLICIES, PROCESSES OR SYSTEMS					
Follow-up of device-related outcomes beyond short-term results done elsewhere	Environmental context and resources	Social-professional role and identity	Recommended behaviour: observability	Health professional cognitions: intention and motivation, Health professional behaviour: nature of the behaviour, Health professional knowledge and skills: knowledge about own practice, Health professional behaviour: self-monitoring or feedback, Professional interactions: referral processes	Yes (professional role or behaviour, observability or knowledge of own behaviour)
Devices implanted not recorded in patient records	Environmental context and resources	–	Incentives and resources: information system	Health professional knowledge and skills: knowledge about own practice, Health professional behaviour: self-monitoring or feedback, Health professional behaviour: capacity to plan change	Yes (resources or information system)
No hospital, national or international systems for AMDE reporting	Environmental context and resources	Knowledge, Reinforcement, Behavioural regulation	Incentives and resources: information system, Incentives and resources: availability of necessary resources, Capacity for organizational change: regulations, rules and policies	Health professional knowledge and skills: domain knowledge, Health professional cognitions: intention and motivation, Health professional behaviour: self-monitoring or feedback, Incentives and resources: non-financial incentives and disincentives, Incentives and resources: quality assurance and patient safety systems, Capacity for organizational change: monitoring and feedback	Yes (resources or information system, knowledge, reinforcement or non-financial incentives or disincentives, regulation or self- or organizational monitoring)
DEVICE MARKET					
Use of specific devices often determined by purchase group contract obligations	Environmental context and resources	–	Health professional behaviour: capacity to plan change	Capacity for organizational change: regulations, rules and policies, Incentives and resources: financial incentives and disincentives, Capacity for organizational change: mandate, authority and accountability, Social, political and legal factors: economic constraints on the health care budget, Social, political and legal factors: contracts	Yes (context and resources or policies, financial incentives and disincentive, authority, budget, contracts)
Lack of responsiveness to AMDEs from industry	Reinforcement	Knowledge, Optimism, Beliefs about consequences, Environmental context and resources	–	Health professional cognitions: expected outcome, Health professional cognitions: intention and motivation, Health professional behaviour: self-monitoring or feedback, Social, political and legal factors: influential people	Yes (reinforcement or feedback or influential people, expected outcome, optimism or motivation)

### Interventions corresponding to TDF domains and TICD determinants

Additional file 2 summarizes the interventions corresponding to TDF domains and TICD determinants selected by one or both mappers.

#### Many interventions were identified

Both frameworks identified numerous interventions for each AMDE reporting theme. For example, the theme *‘AMDEs were considered unexpected or unavoidable’* was mapped by both mappers to the TDF domain of ‘Beliefs about consequences’, for which 23 distinct interventions are suggested across 4 categories (covert learning, comparison of outcomes, natural consequences, and reward and threat). The same theme was mapped by both LD and ARG to the TICD determinant of ‘Health professional cognitions: expected outcome’, for which 2 distinct interventions are suggested (information or educational strategies that provide compelling evidence, and audit and feedback).

Using the TDF, domains selected by both mappers identified a total of 47 unique intervention options across all themes; this included 23 unique interventions to address physician beliefs, 14 unique for organization or system themes, and 35 for device market themes. Using the TICD, determinants selected by both mappers identified 12 unique intervention options, including 2 unique interventions for physician beliefs, 8 for organization or system themes, and 4 for device market themes.

#### Convergence of interventions

As was noted previously, selected domains or determinants were often similar across AMDE reporting themes and determinant levels. Hence, interventions recommended by the TDF and TICD were also similar. For example, across themes describing physician beliefs, interventions frequently recommended by TDF included covert learning, comparison of outcomes, natural consequences, and reward and threat. Common interventions recommended by TICD included information or educational strategies that provide compelling evidence or address reasons for disagreement, audit and feedback, and a local consensus process.

#### Direct relevance of interventions

In some cases, interventions recommended by the TDF and TICD were intuitively linked to the determinant theme. For example, the theme *‘Views about cause of AMDEs confounded by multiple factors’* was mapped to the TDF domain ‘Knowledge’, for which 17 interventions were recommended in the categories of feedback and monitoring and shaping knowledge and natural consequences, which both reflect knowledge sharing. The same theme was mapped to the TICD determinant of ‘Health professional knowledge and skills: domain knowledge’ for which 3 interventions were recommended, including change the mix of professional skills; tailor educational strategies; and disseminate new knowledge, again all focused on knowledge sharing.

In other cases, the applicability of interventions recommended by the TDF and TICD appeared less direct, perhaps owing to a greater degree of complexity in determinants identified in the primary study. For example, at the device market level, the theme *‘Use of specific devices often determined by purchasing group contract obligations’* was mapped to the TDF domain of ‘Environmental context and resources’ for which 14 interventions categorized as antecedents or associations were recommended. These interventions involve restructuring the physical or social environment, or adding or removing prompts or cues, and do not seem to readily address the multi-level restrictions on behaviour of purchasing group contracts. Conversely, mapping the same theme to TICD determinants identified the more granular intervention of improvements in contracts.

Similarly, important themes from the predicate study reflecting complex determinants may not have been well-addressed by either TDF or TICD, leading to less than appropriate interventions. For example, the theme ‘*Views about cause of AMDEs confounded by multiple factors*’ was mapped to the TDF domain of ‘Beliefs about consequences’ by both mappers and the TICD domain of ‘Health professional cognitions: expected outcome’ by both mappers, ultimately leading to 23 corresponding interventions recommended by TDF and 5 recommended by TICD. All of the interventions address knowledge but none appear to fully recognize the interplay of determinants inherent in this theme.

#### Comparison across theoretical frameworks

Overall, although a greater number of TICD determinants were applied across themes and mappers compared with TDF domains, the TDF identified many more unique interventions across all themes (47 for domains selected by both mappers plus additional domains selected by one mapper) compared with the TICD (12 interventions for determinants selected by both mappers plus additional determinants selected by one mapper).

Several interventions recommended by TDF and TICD were similar in meaning, irrespective of the theme. For example, for the physician beliefs theme *‘AMDEs considered expected or unavoidable and not adverse’*, the TDF intervention of comparison of outcomes was conceptually similar to the TICD intervention of audit and feedback, and the TDF intervention of information about health consequences was similar to the TICD intervention of information or educational strategies that provide compelling evidence.

Even when themes were mapped to domains or determinants that were similar in meaning, different interventions were recommended by TDF and TICD in some instances. For example, for the device market theme *‘Lack of responsiveness to AMDEs from industry’*, the TDF interventions (categorized as scheduled consequences) focused on adding or removing rewards, while the TICD interventions (information or educational strategies and audit and feedback) focused on the provision of information.

### Implications for practice

Table 4 summarizes overall study findings and their implications. Knowledge generated by this study addresses the applied objectives of identifying interventions to stimulate AMDE reporting, and comparing the domains or determinants and interventions identified by mapping AMDE reporting themes to the TDF and TICD.

**Table 4:** Summary of findings and implications

Finding	Implication
All AMDE reporting themes mapped to both TDF and TICD	Both theoretical frameworks were useful for systematically analyzing AMDE reporting determinant themes
Multiple TDF domains and TICD determinants were relevant	Provide users with flexibility to choose and further prioritize from among the array of relevant domains/determinants but also raises uncertainty about how many to choose and with what precision
Several TDF domains and TICD determinants chosen by one or both mappers were conceptually similar though labelled differently	Convergence across frameworks could be used to identify a core set of behavioural determinants
Selected TDF domains and TICD determinants chosen by one or both mappers applied to more than one AMDE reporting theme	Convergence within frameworks could be used to identify a core set of behavioural determinants
Domains and determinants selected independently by two mappers often did not match; discrepancy rate similar for TDF and TICD	Selection of TDF domains and TICD determinants may be subjective and influenced by mapper familiarity with a given theoretical framework. It is unclear if a process is needed to resolve discrepancies or, instead, if intervention design should be based on only domains/determinants selected by all independent mappers, or on a core set of domains/determinants most commonly selected by all mappers
Greater number of TICD determinants were applied across themes and mappers compared with TDF domains	Compared with TDF, which focuses on individual level domains, TICD offers multilevel determinants, plus definitions and examples for each, and was thus easier to apply and could be applied with greater precision at a granular level
Numerous interventions corresponded to common TDF domains and TICD determinants selected by both mappers for each AMDE reporting theme	It is unclear how to choose the intervention (s) that are most relevant from among the large number of options presented by the TDF and TICD
Additional interventions corresponded to TDF domains and TICD determinants selected by one mapper	It is unclear if intervention (s) should be chosen based on only those associated with domains/determinants selected by all independent mappers, or with a core set of domains and determinants most commonly selected by all mappers
Given that similar TDF domains and TICD determinants were applied across AMDE reporting themes, corresponding interventions were also convergent	Convergence within frameworks could be used to identify a core set of interventions corresponding to behavioural determinants
Some interventions recommended by TDF and TICD for the same AMDE reporting themes were conceptually similar though labelled differently	Convergence across frameworks could be used to identify a core set of interventions corresponding to behavioural determinants
Although more TICD determinants were applied compared with TDF domains, TDF recommended a greater number of interventions compared with TICD	It is unclear if and how interventions that are most relevant for a given context should be screened or prioritized from among the options recommended by either TDF or TICD
Even when themes mapped to conceptually similar TDF domains and TICD determinants, TDF and TICD often recommended conceptually different interventions	It is unclear how to choose the intervention (s) that are most relevant when two rigorously developed theoretical frameworks differ in the interventions recommended for the same determinant
Some interventions recommended by TICD seemed more intuitively relevant compared with TDF	Compared with TDF, which recommends interventions corresponding to broad domains, TICD recommends interventions corresponding to specific determinants, and may identify interventions that are more relevant. Following the mapping of themes to theoretical frameworks, consultation with stakeholders is likely needed to deliberate the relevance and feasibility of corresponding interventions for a given context.
Complex determinants involving interplay among factors were not well-addressed by TDF or TICD	Domains and corresponding interventions in the TDF or TICD did not fully recognize the complex interplay of determinants inherent in some themes. It is unclear if this is because the frameworks are better suited to exploring determinants in some contexts (i.e. adherence with clinical guideline recommendations) and not others (i.e. reporting of AMDEs.
Neither TDF nor TICD prompt users to prioritize domains or interventions	Neither the TDF nor the TICD prompt users to prioritize among the many potentially applicable domains or interventions as means of limiting or focusing the number and type of interventions

#### Interventions to stimulate AMDE reporting

AMDE reporting themes were mapped by both mappers to several domains and determinants, which identified corresponding interventions common to the TDF and TICD. Information or educational strategies that provide evidence about AMDEs, and audit and feedback of AMDE-related data were identified as interventions to target physician beliefs; improve information systems, and reminder cues, prompts and awards were identified to target organization or system themes; and modify financial/non-financial incentives, and share data on outcomes associated with AMDEs were identified to address device market themes. However, issues and discrepancies in the application of TDF and TICD raise uncertainty about which or how many interventions may be relevant to promote and support AMDE reporting.

#### Application of the TDF and TICD

Issues revealed by this study include a lack of clarity about how directly relevant domains or determinants should be and therefore which and how many to select; if and how to resolve discrepancies in the selection of domains or determinants across multiple mappers; and how to choose interventions from among the large number associated with selected domains and determinants. Several TDF domains and TICD determinants were relevant, similar in meaning, and selected by both mappers. Convergence within and across TDF and TICD identified a core set of behavioural determinants and corresponding interventions. Thus, both theoretical frameworks were useful for selecting behavioural determinants to which AMDE reporting themes matched and corresponding interventions.

However, TDF domains and TICD determinants selected independently by both mappers often did not match, and a large number of interventions corresponded to the TDF domains and TICD determinants selected by one or both mappers. Even when themes mapped to TDF domains and TICD determinants with similar definitions, the frameworks often recommended different interventions. TICD recommended interventions that seemed to be more directly applicable to a behavior such as AMDE reporting with multi-level determinants as compared with the TDF. Domains and corresponding interventions in the TDF or TICD did not fully recognize the complex interplay of determinants inherent in some themes; it is unclear if this is because the frameworks are better suited to exploring determinants in some contexts (i.e. adherence with clinical guideline recommendations) and not others (i.e. reporting of AMDEs.

Discrepancies in applying TDF and TICD may be accounted for by distinctions between their content and format. TDF includes determinant domains largely focused on the individual level while TICD includes determinant domains and determinants spanning multiple levels and, unlike TDF, offered definitions and examples to guide the application of these more granular determinants. Although more TICD determinants were applied compared with TDF domains, TDF recommended a greater number of interventions compared with TICD. While the predicate study did not itself prioritize determinants, neither the TDF nor the TICD prompt users to prioritize among the many potentially applicable domains or interventions as means of limiting or focusing the number and type of interventions. Overall, uncertainty remains about the optimal way to identify interventions that match behavioural determinants for a given behaviour, and the precision and relevance of those choices.

## Discussion

This study was a naturalistic application of the TDF and TICD to identify evidence-based interventions corresponding with known determinants of AMDE reporting and, in so doing, to explore how use of these theoretical frameworks could be optimized. Both TDF and TICD were useful in identifying several interventions that could promote and support AMDE reporting. However, it is uncertain which interventions are the best options given discrepancies in the selection of TDF domains and TICD determinants, and corresponding interventions across theoretical frameworks and independent mappers. The content and format of TICD (well-defined domains and determinants spanning individual, organizational, system and environmental levels) may make it easier to apply than the TDF for individuals who are not familiar with either framework. Even still, uncertainty remains about how to best apply the frameworks in practice and their precision when used to design behaviour change interventions.

Our findings align with previous work highlighting the uncertainty and challenges surrounding the application of theoretical frameworks to design behaviour change interventions. Lipworth et al. analyzed determinants of the uptake of clinical quality interventions and found that all 14 TDF domains and numerous corresponding interventions were relevant, necessitating a “drilling down” to identify those that were most “contextually salient” [25]. Lawton et al. used the TDF to conduct and analyze the findings of 60 interviews with 60 general practice health care professionals regarding adherence to various clinical recommendations [3]. A wide variety of determinants were identified but it was difficult to “pinpoint which determinants, if targeted by an implementation strategy, would maximize change”, underscoring the need for “broader contextual consideration”. One potential explanation is reality that theoretical frameworks do not address causal mechanisms, or how change occurs, which presents a challenge when attempting to identify which intervention (s) are most likely to support improvement [1]. Phillips et al. interviewed 10 health care professionals from six disciplines who used the TDF [26]. Frequently cited challenges experienced when applying it included the time and resources required to use the TDF, lack of clear operational definitions, and overlap between domains. Participants found it difficult, complicated, unwieldy, and subjective to interpret and apply the domains [26]. Birken et al. conducted a systematic review of five protocols and seven studies that used both the TDF and the Consolidated Framework for Implementation Research (CFIR) to examine the rationale for having applied both frameworks [27]. Authors of included studies justified the use of both frameworks by stating that one offered greater insight on determinants and the other on interventions, although which framework offered determinants versus interventions was interchangeable across studies. A conceptual analysis of reasons for the failure of interventions designed based on the TICD offered several reasons including potential mismatch of determinants to interventions or a subsequent mismatch of interventions to targeted groups and settings [6]. Thus, our research and that of others reveals uncertainty and challenges in the application of theoretical frameworks to design behaviour change interventions. More recently a guide to use of the TDF was published [28]. The guide specifies that coding disagreements could be resolved by either consensus among coders or assessment of inter-rater reliability, and when uncertain about coding to apply all relevant TDF domains. However, these suggestions do not help users select from among the many potential interventions identified by this approach.

The interpretation and application of these findings may be limited by several factors. Independent mappers made a deliberate decision to not coordinate their interpretation of the TDF and TICD before mapping, nor did they intend to discuss and resolve discrepancies after mapping. The objective was to independently apply the theoretical frameworks specifically to explore the nature of any arising discrepancies as a means of identifying problems that may be encountered by others when employing these tools in implementation planning. Each mapper had differing levels of familiarity with both frameworks, thereby precluding the ability to comment on the nature of discrepancies when those applying the framework have similar levels of experience. As ARG conducted the interviews for the primary study, it is possible her familiarity with the data may have led to a contextual advantage when applying the frameworks. The challenges and discrepancies encountered when applying the frameworks may be specific to the single case examined, that of determinants of AMDE reporting. Also, the TDF and TICD may be better suited to assessing determinants and corresponding interventions for some contexts more so than others; that could not be determined by this study and will require future research.

With respect to selecting determinants and interventions, our research and that of others [3, 6, 25–27] found that the TDF and TICD are useful for fully describing the range of potentially relevant determinants, a task perhaps best done by implementation scientists who are familiar with the constructs and their definitions. This suggests that selecting the most relevant determinants and interventions is likely to benefit from collaboration with stakeholders with context-specific knowledge. Processes such as Intervention Mapping, whereby researchers and health care professionals can jointly choose and design interventions based on the identification and prioritization of determinants, may prove useful for developing and evaluating interventions that are more likely to improve the delivery and outcomes of care [29].

Further research applying the TDF and TCID in specific contexts is needed in order to resolve the differences between them and clarify the circumstances for which each framework is most useful. The critical need remains to make these tools easier to use for a broader audience, and to establish a reliable way to identify which of many potential interventions are likely to successfully address specific determinants. Key considerations include how many independent mappers are needed, what process is needed to resolve discrepancies across mappers, whether intervention design should be based on only those domains or determinants selected by all independent mappers, or on some other combination of domains or determinants identified; and how best to prioritize the selection of potential interventions. Further insight or framework development is also needed to help users address complex determinants, and to prioritize domains and corresponding interventions.

## Conclusions

The TDF and TICD were employed to identify behavioural interventions corresponding to determinants of the reporting of AMDEs. Interventions common to both frameworks included information or educational strategies that provide evidence about AMDEs; audit and feedback of AMDE data; improved information systems; reminder cues, prompts and awards; modifying financial/non-financial incentives; sharing data on outcomes associated with AMDEs. Challenges and discrepancies in the application of frameworks raise uncertainty about which or how many interventions may be relevant to promote and support AMDE reporting. Given the worldwide imperative to promote the use of evidence-based innovations and improve the quality and safety of care, there is an urgent need to make tools such as the TDF and TICD easier to use for a broader audience, and to establish a reliable way to identify which of many potential interventions are likely to successfully address specific determinants. Just as research more broadly has seen a shift from the production and dissemination of evidence to the implementation of evidence, and it is time for the field of implementation science to shift from the development of frameworks to supporting their application in practice.

## Additional files

Additional file 1: AMDE reporting themes that emerged from previous study [14] (DOCX 17 kb).

Additional file 2: Comparison of interventions identified across theoretical frameworks (DOCX 30 kb).

## Abbreviations

AMDE: Adverse medical device event
ARG: Anna R Gagliardi
LD: Laura Desveaux
TDF: Theoretical Domains Framework
TICD: Tailored Implementation for Chronic Diseases
