# Supplementary material for: Comparing the application of two theoretical frameworks to describe determinants of adverse medical device event reporting: secondary analysis of qualitative interview data
Source: BMC Health Serv Res. 2018 Jun 4;18:402. doi: 10.1186/s12913-018-3251-2 (PMC5987566; doi:10.1186/s12913-018-3251-2)
Supplement: Supplementary file 1 — AMDE reporting themes that emerged from previous study [14] (DOCX 17 kb). [file 12913_2018_3251_MOESM1_ESM.docx]

Additional File 1. AMDE reporting themes that emerged from previous study [14]

| AMDE reporting themes | Exemplar quotes |
| --- | --- |
| PHYSICIAN BELIEFS | |
| AMDEs considered expected or unavoidable and not adverse unless outcomes catastrophic; viewed as more severe in other specialties | It’s something that we all recognize as a complication or a risk of putting a large foreign device in someone’s chest (04CE)  I’m sure in cardiovascular surgery they have had more incidents that can happen with their devices. For us it just means take it out and put a new one in (07OL) |
| AMDEs within 2 years of use were considered unusual | Typically it would occur in the months and early years following the procedure where you would expect such an implant to last five, ten or 15 years (03OL) |
| Views about cause of AMDEs confounded by multiple factors | It’s hard to know if it’s the device itself, the way the operator used it, or the way the patient’s anatomy might have changed over time (19CM) |
| Incidence of AMDEs has decreased, thus devices were thought to be improved | That happens from time to time but as the devices have become smaller and new devices have come out the incidence of that has clearly dropped off (04CE) |
| POLICIES, PROCESSES or SYSTEMS | |
| Follow-up of device-related outcomes beyond short-term results done elsewhere | The only real monitoring that goes on is we put something in, we follow it up to see whether it healed and the problem went away, so they’re followed specifically for the implant, they’re just followed by us for the patient in general (12OM) |
| Devices implanted not recorded in patient records | It was actually a whole lot of leg work on our part. The company sends out the list of batch numbers that were affected and we had to go through all of our patient records to find out whether anybody had it. We have a stack of notebooks with patient information and stickers from the product so we had to keep going through it and looking for the lot numbers back through the period of time the company says that the lot number was produced (12OM) |
| No hospital, national or international systems for AMDE reporting | I’m not sure that there actually is a process to be very honest (21CM) |
| DEVICE MARKET | |
| Use of specific devices often determined by purchase group contract obligations | Sometimes the implant you put in is not what you think is the best for the patient because that’s the only thing available through the buying group (07OL) |
| Lack of responsiveness to AMDEs from industry | Eventually the manufacturer paid attention and then changed the engineering of the device. But that took probably somewhere between two and three years. They’ve invested a huge amount of money in product development and then they’ve got a big back inventory. So if there’s a big cost associated with change, and people have figured out a work around, then there’s a lot less pressure on the company to change (06OM) |

O orthopedic C cardiovascular E early career M mid-career L late career
