# Supplementary material for: Comparing the application of two theoretical frameworks to describe determinants of adverse medical device event reporting: secondary analysis of qualitative interview data
Source: BMC Health Serv Res. 2018 Jun 4;18:402. doi: 10.1186/s12913-018-3251-2 (PMC5987566; doi:10.1186/s12913-018-3251-2)
Supplement: Supplementary file 2 — Comparison of interventions identified across theoretical frameworks (DOCX 30 kb). [file 12913_2018_3251_MOESM2_ESM.docx]

Additional File 2. Comparison of interventions identified across theoretical frameworks

| **Determinant themes from AMDE study** | **TDF interventions** | | **TICD interventions** | | **Apparent match in underlying meaning** |
| --- | --- | --- | --- | --- | --- |
|  | **Domains selected by both mappers** | **Domains selected by one mapper** | **Domains:determinants selected by both mappers** | **Domains:determinants selected by one mapper** |  |
| **PHYSICIAN BELIEFS** |  |  |  |  |  |
| AMDEs considered expected or unavoidable and not adverse unless outcomes catastrophic; viewed as more severe in other specialties | - Covert learning (3)   - imaginary punishment   - imaginary reward   - vicarious consequences - Comparison of outcomes (3)   - credible source   - pros and cons   - comparative imagining of future outcomes - Natural consequences (6)   - information about health consequences   - salience of consequences   - information about social and environmental consequences   - monitoring of emotional consequences   - anticipated regret   - information about emotional consequences - Reward and threat (11)   - material incentive (behaviour)   - material reward (behaviour)   - non-specific reward   - social reward   - social incentive   - non-specific incentive   - self-incentive   - incentive (outcome)   - self-reward   - reward (outcome)   - future punishment | [TDF does not suggest interventions for one domain selected by one mapper] | - Information or educational strategies that provide compelling evidence - Audit and feedback | - Educational strategies that address the reasons for disagreement - A local consensus process | - Comparison of outcomes and Audit and feedback - Natural consequences – information about health consequences and Information or educational strategies that provide compelling evidence |
| AMDEs within 2 years of use were considered unusual | - Covert learning (3)   - see above - Comparison of outcomes (3)   - see above - Natural consequences (6)   - see above - Reward and threat (11)   - see above | [TDF does not suggest interventions for one domain selected by one mapper] | - Information or educational strategies that provide compelling evidence - Audit and feedback | - Educational strategies that address the reasons for disagreement - A local consensus process | - Comparison of outcomes and Audit and feedback - Natural consequences – information about health consequences and Information or educational strategies that provide compelling evidence |
| Views about cause of AMDEs confounded by multiple factors | --- | - Covert learning (3)   - see above - Comparison of outcomes (3)   - see above - Natural consequences (6)   - see above - Reward and threat (11)   - see above - Feedback and monitoring (7)   - monitoring of behaviour by others without feedback   - feedback on behaviour   - self-monitoring of behaviour   - self-monitoring of outcome(s) of behaviour   - monitoring of outcome(s) of behaviour without feedback   - biofeedback   - feedback on outcome(s) of behaviour - Shaping knowledge (4)   - instruction on how to perform the behaviour   - information about antecedents   - re-attribution   - behavioural experiments | --- | - Educational strategies that address the reasons for disagreement - A local consensus process - Change the mix of professional skills in the targeted teams or organizations - Tailor educational strategies to the level of expertise of the targeted healthcare professionals - Targeted dissemination of new knowledge | --- |
| Incidence of AMDEs has decreased, thus devices were thought to be improved | --- | - Covert learning (3)   - see above - Comparison of outcomes (3)   - see above - Natural consequences (6)   - see above - Reward and threat (11)   - see above - Self-belief (2)   - verbal persuasion about capability   - mental rehearsal of successful performance   - focus on past success   - self-talk | - Information or educational strategies that provide compelling evidence - Audit and feedback | - Educational strategies that address the reasons for disagreement - A local consensus process | - Comparison of outcomes and Audit and feedback - Natural consequences – information about health consequences and Information or educational strategies that provide compelling evidence |
| **Sub-total unique or matching interventions** | **23** | **36** | **2** | **5** |  |
| **POLICIES, PROCESSES OR SYSTEMS** |  |  |  |  |  |
| Follow-up of device-related outcomes beyond short-term results done elsewhere | - Antecedents (6)   - restructuring the physical environment   - restructuring the social environment   - avoidance/reducing exposure to cues for the behaviour   - distraction   - adding objects to the environment   - body changes - Associations (8)   - prompts/cues   - cue signaling reward   - reduce prompts/cues   - remove access to the reward   - remove aversive stimulus   - satiation   - exposure   - associative learning | --- | Provide feedback, information or education regarding the benefits of adhering to the recommendation | - Local discussion and consensus - Discuss resistance - Provide good arguments why adherence is important - Involve opinion leaders - Strategies that are tailored to the stage of change of individuals in the targeted health care professional - Audit and feedback - Modification of the context to prompt the new behavior, i.e. reminders, changes in layout or equipment - Monitoring, feedback, reminder systems - Integration of the recommendation into routine care plans or local protocols - Provide necessary resources, support or rewards - Structured referral sheets - Involvement of consultants in primary care educational activities - Tailored patient information - Patient-held medical records | - Associations – prompts, cues and Modification of the context - Associations – prompts, cues and Monitoring, feedback reminder systems - Associations – cue signaling rewards and Provide necessary resources, support or rewards |
| Devices implanted not recorded in patient records | - Antecedents (6)   - see above - Associations (8)   - see above | --- | - Improve the information system - Provide appropriate incentives to record needed information | - Audit and feedback - Extra resources, support or aids to assist with planning necessary changes - Provide feasible objectives for change, an inventory of typical problems and solutions - Monitoring, feedback, reminder systems - Integration of the recommendation into routine care plans or local protocols - Provide necessary resources, support or rewards | - Antecedents – restructure the physical environment and Improve the information system - Associations – cue signaling reward and Provide appropriate incentives; Provide necessary resources, support or rewards - Associations – prompts or cures and Provide feasible objectives for change; Monitoring, feedback, reminder systems |
| No hospital, national or international systems for AMDE reporting | - Antecedents (6)   - see above - Associations (8)   - see above | - Feedback and monitoring (7) - Shaping knowledge (4) - Natural consequences (6) - Scheduled consequences (10) - Reward and threat (11) | - Improve the information system - Provide appropriate incentives to record needed information - Provide the necessary resources - Reduce the need for additional resources or their cost - Task shifting - Change the regulations, rules or policies - Make adaptations to enable adherence to the recommendation within the existing regulations | - Monitoring, feedback, reminder systems - Integration of the recommendation into routine care plans or local protocols - Provide necessary resources, support or rewards - Remove or modify disincentives - Provide incentives - Establish or improve a quality assurance or patient safety system - Adapt implementation strategies to work within the constraints of the existing systems - Use external support to provide monitoring and feedback - Establish required monitoring and feedback | - Reward and Threat; Associations – prompts, cues and Provide appropriate incentive; Monitoring, feedback, reminder systems - Reward and Threat; Associations – cue signaling reward and Provide necessary resources, support or rewards; Provide incentives - Associations – remove aversive stimulus and Remove or modify disincentives - Shaping knowledge – instructions to perform behaviour and Integration of recommendations in care plans/protocols - Feedback and monitoring – feedback on behaviour or outcomes of behaviour and Use external support to provide monitoring and feedback; Establish required monitoring and feedback |
| **Sub-total unique or matching interventions** | **14** | **38** | **8** | **22** |  |
| **DEVICE MARKET** |  |  |  |  |  |
| Use of specific devices often determined by purchase group contract obligations | - Antecedents (6)   - see above - Associations (8)   - see above | --- | - Extra resources, support or aids to assist with planning necessary changes - Provide feasible objectives for change, an inventory of typical problems and solutions | - Remove or modify the financial disincentive - Provide financial incentives - Change the regulations, rules or policies - Make adaptations to enable adherence to the recommendation within the existing regulations, rules and policies - Allocate or reallocate authority to make relevant decisions - Monitor organisational changes - Formally appoint implementation leaders - Use external change agents - Formal agreements regarding accountability - Increase transparency - Pragmatic consideration of what is feasible - Gradual change - Shifting resources from elsewhere in the healthcare budget - Increasing the healthcare budget - Improvements in contracts, including provision for enforcement | - Associations – prompts, cues and Provide feasible objectives for change - Associations – remove aversive stimulus and Remove or modify financial disincentive - Associations – cue signaling reward and Provide financial incentives |
| Lack of responsiveness to AMDEs from industry | - Scheduled consequences (10)   - behaviour cost   - punishment   - remove reward   - reward approximation   - rewarding completion   - situation-specific reward   - reward incompatible behaviour   - reward alternative behaviour   - reduce reward frequency   - remove punishment - Reward and threat (11)   - see above | - Feedback and monitoring (7)   - see above - Shaping knowledge (4)   - see above - Natural consequences (6)   - see above - Self-belief (2)   - see above - Covert learning (3)   - see above - Comparison of outcomes (3)   - see above - Reward and threat (11)   - see above - Antecedents (6)   - see above - Associations (8)   - see above | - Information or educational strategies that provide compelling evidence - Audit and feedback | - Local discussion and consensus - Discuss resistance - Provide good arguments why adherence is important - Involve opinion leaders - Strategies that are tailored to the stage of change of individuals in the targeted healthcare professional - Monitoring, feedback, reminder systems - Integration of the recommendation into routine care plans or local protocols - Engage and persuade opponents - Engage and capitalize on supporters - Facilitate dialogue, understanding of conflicting perspectives and achievement of a consensus | - Natural consequences – information about health consequences and Information or educational strategies that provide evidence - Natural consequences – salience of consequences and Provide good arguments why adherence is important - Feedback and monitoring – feedback on behaviour or outcomes of behaviour and Audit and feedback; Monitoring, feedback, reminder systems - Shaping knowledge – instruction on how to perform the behaviour and Integration of recommendations into care plans/protocols - Self-belief – verbal persuasion about capability and Engage and persuade opponents |
| **Sub-total unique or matching interventions** | **35** | **56** | **4** | **25** |  |
| **Total unique or matching interventions** | **47** | **61** | **12** | **45** |  |
